# Extravasation of iodinated contrast medium in cancer patients undergoing computed tomography

**DOI:** 10.1590/0100-3984.2017.0064

**Published:** 2018

**Authors:** Hernandes Cerqueira de Souza Silva, Almir Galvão Vieira Bitencourt, Rubens Chojniak

**Affiliations:** 1 MSc, Head Nurse in the Imaging Department of A.C.Camargo Cancer Center, São Paulo, SP, Brazil.; 2 MD, PhD, Radiologist in the Imaging Department of A.C.Camargo Cancer Center, São Paulo, SP, Brazil.; 3 MD, PhD, Radiologist, Director of the Imaging Department of A.C.Camargo Cancer Center, São Paulo, SP, Brazil.

**Keywords:** Tomography, X-ray computed, Contrast media, Extravasation of diagnostic and therapeutic materials, Medical oncology

## Abstract

**Objective:**

The aim of this study was to evaluate the incidence of extravasation of iodinated contrast medium (ICM) at the site of intravenous injection in oncology patients submitted to computed tomography (CT).

**Materials and Methods:**

This was a retrospective, descriptive, single-center study that evaluated all patients who underwent CT with ICM administration and presented ICM extravasation, at a cancer center, between January 2010 and December 2015.

**Results:**

During the study period, we evaluated a total of 99,076 ICM injections and identified 199 cases of extravasation, the incidence rate therefore being 0.20%. Among the patients who presented extravasation, the mean age was 59.22 years and 60% were female. The extravasation was classified as mild in 94.10% of the patients and as moderate in 5.90%. There were no cases of severe extravasation in the sample.

**Conclusion:**

The incidence of ICM extravasation in cancer patients submitted to CT in the present study was similar to that reported for the general population, according to other studies in the literature. The vast majority of cases of extravasation were considered mild, and no severe cases were observed in the study sample.

## INTRODUCTION

It is estimated that 80 million computed tomography (CT) scans were performed in the United States in 2010, and an annual increase of 10% is projected^1^. Most procedures are performed with iodinated contrast medium (ICM) injection. However, as with any medicine, the use of ICM is not entirely risk-free.

Even with correct application of the venipuncture technique, extravasation of ICM occurs and is a multifactorial event. Extravasation is defined as inadvertent delivery of vesicant fluid into healthy surrounding tissue rather than into the intended vessel, potentially reaching adjacent structures such as subcutaneous, nerve, and muscle tissue^2^.

The rate of occurrence of extravasation is relatively high. In the majority of cases however, lesions are self-limiting and recede after intervention of the diagnostic imaging team. The reported incidence of extravasations ranges from 0.25% to 1.2%^3-5^. A review of the literature demonstrated that prevention is the only way to decrease the rates and complications of ICM extravasation.

Cancer patients are exposed to ICM extravasation, because most of the known risk factors are present in the cancer treatment routine. Among such risk factors are treatments that sensitize the venous network, such as chemotherapy, and the need for multiple punctures. However, in the literature, we found no studies specifically evaluating the incidence of and risk factors associated with extravasation of contrast medium in cancer patients submitted to CT with intravenous administration of ICM.

The objectives of this study were to evaluate the incidence of ICM extravasations in patients undergoing CT, as well as the demographic, clinical, oncologic, and clinical characteristics of those patients.

## MATERIALS AND METHODS

This was a retrospective, descriptive, single-center study of all patients who underwent CT with intravenous contrast at a referral center for cancer and then presented extravasation, between January 2010 and December 2015. A database of the daily routine at the institution was used in order to identify contrast medium extravasation events. Before data collection had begun, the study was approved by the research ethics committee of the institution.

The exams were performed in a Philips Big Bore 16-slice device (Philips Medical Systems; Cleveland, OH, USA). The ICM used was low-osmolarity Optiray (Mallinckrodt; Raleigh, NC, USA)-ioversol at 678 mg/mL; organically bound iodine: 320 mg/mL-in a syringe filled with 75 mL, 100 mL, or 125 mL. ICM injections were performed using an Optistar Elite contrast injector (Covidien; Dublin, Ireland), the MCI being preheated to 37ºC in an oven.

For classification of the severity of the ICM extravasation events, we used the classification system suggested by Tardáguila de la Fuente et al.^6^, as described below.

- Mild: Absence of initial symptoms, presenting only pain, inflammation, mild erythema, and mild or moderate edema, resolved with limb elevation and local application of cold.
- Moderate: Moderate or severe erythema, presence of vesicles, persistence of pain, and inflammation or lesions requiring additional treatment. All symptoms resolved within two weeks.
- Severe: Serious adverse effects; cyanosis, tissue necrosis, or symptoms lasting more than two weeks, such as persistent pain and edema, difficulty in locomotion, and requiring surgery.

The treatment protocol for ICM extravasation events is based on the international recommendations of the American College of Radiology, and the following steps are taken: clinical evaluation by the radiologist; clinical evaluation of the radiology nurse; withdrawal of venous catheter; estimated volume extravasated; local application of an ice pack in the examination room, when possible; and rest with elevation of the affected limb. For patients with an extravasation volume greater than 100 mL and clinical signs of progression, such as persistent pain or cyanosis of the affected limb, a vascular surgery consult is requested or the patient is referred to the emergency department.

The extravasation rate was calculated as follows: total number of extravasation cases, divided by the number of exams involving the use of ICM in the period, multiplied by 100. For statistical analysis, we used the IBM SPSS Statistics software package, version 23.0 (IBM Corporation; Armonk, NY, USA). The chi-square test or the Fisher’s exact test was used in order to associate categorical variables with the severity of ICM extravasation. Continuous variables were assessed by unpaired Student’s t-test. The significance level adopted was 5%, and the results considered statistically significant were those with a *p*-value less than or equal to 0.05.

## RESULTS

### Incidence of extravasation

In the period from 2010 to 2015, a total of 99,076 ICM injections were administered in the CT sector. In that period, 199 ICM extravasations were recorded: 185 (92.96%) in patients with a diagnosis of cancer and 11 (5.53%) in patients without. For 3 patients (1.51%), there were missing data, and those patients were therefore excluded from the descriptive analysis. The incidence rate of ICM extravasation calculated was 0.2008%. Table 1 shows the annual distribution of cases of extravasation.

**Table 1:** Annual distribution of the incidence of iodinated contrast agent extravasation in cancer patients undergoing computed tomography.

Event year	Total occurrences*	Number of exams	Incidence rate
2010	10	11,562	0.0864%
2011	15	12,894	0.1163%
2012	26	15,870	0.1638%
2013	49	17,626	0.2779%
2014	44	19,528	0.2253%
2015	55	21,596	0.2546%

* N = 199 patients: 185 with cancer, 11 without cancer, 3 undetermined.

### Demographic and clinical characteristics

For the characterization of the demographic and clinical characteristics, we evaluated 185 patients diagnosed with cancer.

The mean age for the patient group was 59.22 years, with a standard deviation of 15.18 years. The median age was 61 years, with a 25-75% interquartile range of 50.0-70.5 years. The minimum age was 11 years, and the maximum age was 89 years. The gender distribution of the patients was as follows: 74 (40%) were male; and 111 (60%) were female.

Body mass index measurements presented a mean of 25.87 kg/m^2^ and a median of 25.00 kg/m^2^. Among the diagnoses of cancer, breast neoplasms were the most common, with 28 cases, followed by malignant lung neoplasms, with 19. Of the sample, 70.90% were receiving, or had previously received, chemotherapy, whereas only 44.90% had received radiotherapy. Regarding the clinical stage, 42 (22.70%) of the patients in the sample were classified as clinical stage III and 89 (48.10%) were classified as clinical stage IV. Regarding the origin of the patient at the time of the extravasation event, 136 were outpatients, 37 were hospitalized, 8 had been referred from the emergency department, and 4 were of unknown provenance.

### Characteristics of extravasation and clinical evolution

For the evaluation of the characteristics of extravasation and clinical evolution, we evaluated data related to 185 patients diagnosed with cancer.

In the present study, the mean extravasated ICM volume was 39.13 mL, with a median of 30.00 mL. The venous catheter most often used was a 22-gauge catheter, which was employed in 120 (64.90%) of the patients, followed by a 24-gauge catheter, which was employed in 47 (25.40%). The infusion rate at the time of extravasation was 1 mL/s in 19 patients (10.30%), 2 mL/s in 109 (58.90%), and 3 mL/s in 10 (5.40%).

The injection was given into the right upper arm in 93 patients (50.30%) and into the left upper arm in 80 (43.20%). The puncture site was the antecubital fossa in 130 patients (70.30%), the forearm in 25 (13.50%), and the back of the hand in 9 (4.90%). The edema at the extravasation site was considered mild in 80 patients (48.78%), moderate in 65 (39.63%), and intense in 19 (11.58%). We observed hyperemia in 61 cases (33.00%) and pain in 64 (34.60%).

After ICM extravasation, 25 patients (13.50%) were referred to the emergency department for evaluation or were evaluated in the imaging department by the vascular surgery team. However, none of the extravasation events evolved to complications that required hospitalization or surgery. The remaining 157 patients (84.90%) did not require evaluation by other specialists.

After the extravasation event, 129 patients (69.70%) remained in the radiology unit for up to 1 h, 42 (22.70%) remained for 1-2 h, and 6 (3.20%) remained longer than 2 h. Of the patients in the sample, 2 (1.10%) had a history of ICM extravasation, whereas 180 (97.30%) did not.

The extravasation was classified as mild in 174 (94.10%) of the patients and as moderate in 11 (5.90%). None of the cases of ICM extravasation were classified as severe. In comparison with the patients who had mild extravasation, those with moderate extravasation had a higher mean body mass index (*p* = 0.009) and a higher extravasation volume (*p* < 0.001) as detailed in Table 2. The intensity of extravasation did not show a statistically significant association with age, gender, patient provenance, reason for the examination, clinical stage, chemotherapy, radiotherapy, venous puncture site, and the volume of contrast administered (Table 3).

**Table 2:** Evaluation of differences between mild and moderate risk of ICM extravasation in cancer patients undergoing computed tomography.

Variable	Risk	N	Mean	Standard deviation	Standard error of the mean	*P*-value
Age at occurrence	Mild	175	59.21	15.32	1.158	0.99
	Moderate	10	59.30	13.29	4.203	
Body mass index	Mild	170	25.60	5.45	0.41800	0.009*
	Moderate	10	30.39	7.74	2.44647	
Contrast volume	Mild	131	95.99	21.82	1.906	0.21
	Moderate	10	105.00	21.86	6.912	
Extravasation volume	Mild	156	36.96	25.61	2.051	< 0.001*
	Moderate	9	76.67	13.23	4.410	

Differences between means considered significant at *p* < 0.05. Student's t-test for equality of means.

**Table 3:** Correlation between clinical and demographic variables with the degree of extravasation of ICM in cancer patients submitted to computed tomography.

		Degree of extravasation Mild Moderate					
		Mild			Moderate		Pearson's chi-square test
Variable		N	%		N	%	*p* < 0.05
Gender	Masculine	71	95.9		3	4.1	*p* = 0.742
	Feminine	104	93.7		7	6.3	
Body mass index classification	Not available	5	100.0		0	0.0	*p* = 0.673
	Underweight	15	100.0		0	0.0	
	Obese	35	92.1		3	7.9	
	Normal weight	71	95.9		3	4.1	
	Overweight	49	92.5		4	7.5	
Patient provenance	Outpatient	127	93.4		9	6.6	*p* = 0.511
	Inpatient	36	97.3		1	2.7	
	Emergency	8	100.0		0	0.0	
Reason for examination	Screening	2	100.0		0	0.0	*p* = 0.940
	Diagnosis	12	100.0		0	0.0	
	Staging	19	86.4		3	13.6	
	Response evaluation	27	90.0		3	10.0	
	Follow-up	91	98.9		1	1.1	
	Other	21	87.5		3	12.5	
Chemotherapy	No	48	90.6		5	9.4	*p* = 0.202
	Current	69	94.5		4	5.5	
	Previous	57	98.3		1	1.7	
Radiotherapy	No	95	93.1		7	6.9	*p* = 0.341
	Yes	79	96.3		3	3.7	
Clinical stage	Stage I	13	92.9		1	7.1	*p* = 0.651
	Stage II	9	100.0		0	0.0	
	Stage III	41	97.6		1	2.4	
	Stage IV	84	94.4		5	5.6	
	Data unavailable	28	90.3		3	9.7	
Caliber of venous catheter used	Catheter 24G	45	95.7		2	4.3	*p* = 0.863
	Catheter 22G	113	94.2		7	5.8	
	Catheter 20G	8	88.9		1	11.1	
	Catheter 18G	1	100.0		0	0.0	
Puncture site	Antecubital fossa	123	94.6		7	5.4	*p* = 0.890
	Forearm	24	96.0		1	4.0	
	Back of the hand	9	100.0		0	0.0	
	Other sites	1	100.0		0	0.0	

## DISCUSSION

The incidence of ICM extravasation in the present study was 0.20%, with a mean of 0.19%, a median of 0.19%, and a 25-75% interquartile range of 0.11-0.26%. The cancer patients who presented extravasation were heterogeneous in terms of their clinical and demographic characteristics, cases of extravasation occurring regardless of gender, age group, body mass index, the presence of neoplasms, clinical stage, and type of treatment, showing that any patient can present this type of complication. The mean volume of extravasated ICM was 39.13 mL. The severity of the event was classified as mild in 174 (94.10%) of the cases and as moderate in 11 (5.90%). None of the cases of ICM extravasation were classified as severe. None of the patients in the study sample developed morbidity related to the extravasation event.

A study by Dykes et al., compiled a large database of information collected between February 2009 and December 2013 from 58 radiology centers in the United States, for a total of 1085 extravasations, demonstrated an extravasation rate of 0.24%, with a median of 0.21% and a 25-75% interquartile range of 0.12-0.31%^7^. In 2014, Shaqdan et al. published the results of a study conducted at the Harvard Medical School Massachusetts General Hospital in the United States, reporting an incidence rate of 0.13%. The retrospective data were collected from June 2008 to June 2013 and referenced a total of 451 extravasations in 352,125 procedures. That rate was lower than the 0.20% found in the present study, although those authors reported some limitations. For example, the data collection was performed electronically at several diagnostic centers, resulting in missing and incomplete data. Another factor that might have affected the calculation of the extravasation rate was that patients with an extravasation volume of less than 10 mL were excluded^8^. In 2007, Wang et al. published the results of a study of 69,657 injections of ICM and 475 events of extravasation in adult and pediatric patients, translating to an incidence rate of 0.70%, with extravasated volumes of 3-150 mL. In the present study, the main symptoms observed were edema and pain. Evaluation by a plastic surgeon was indicated in 38 adults and 6 children. Additional treatment was required in 7 adults and 1 child. One adult patient evolved to compartment syndrome after extravasation of 75 mL of contrast medium in the back of the hand, and one pediatric patient developed brachial plexopathy after extravasation of 18 mL in the arm^9^. Wang et al. reported a higher rate than that found in our sample. However, their study began more than 15 years ago, and we believe that the practices related to the identification and management of patients at higher risk for extravasation have improved since then, influencing the current incidence rate^9^.

The incidence of ICM extravasation demonstrated in our study is within the range reported in the literature. Because we studied a population of patients in cancer treatment, some interventions cited as risk factors (e.g., chemotherapy, multiple venous punctures, and lymphadenectomy) are present in this population. However, the incidence rate in our sample was below those reported in a few studies that assessed general populations of patients with other diseases, such as heart disease. In our study, the majority of patients presenting ICM extravasation (60%) were female. Wang et al. and Shaqdan et al. reported higher rates of extravasation in female patients, suggesting that women are at greater risk for extravasation of contrast media^8,9^.

Cohan et al. and Bellin et al. cited undergoing chemotherapy as a risk factor for extravasation^10,11^. In the present study, 131 (70.80%) of the patients were undergoing or had undergone chemotherapy. Because we evaluated a group of cancer patients, this proportion of patients with a history of chemotherapy is expected, given that chemotherapy is one of the pillars of cancer treatment. We found that the rate of extravasation was not affected by the chemotherapy status.

In the present study, 70.81% of the patients who had ICM extravasation were classified as having clinical stage III or IV disease. However, in the literature, we found no studies including clinical stage evaluations with which we could compare our findings.

The majority of patients in our study sample were outpatients. However, we did not have data from the entire population, which would have allowed us to determine whether the incidence of extravasation was higher among outpatients. Shaqdan et al. reported a significant difference between inpatients and outpatients in terms of the incidence of extravasation, which was higher in the former^8^.

In the present study, the mean volume of extravasated ICM was 39.13 mL (range, 2-110 mL). We categorized the extravasated volumes for comparison with a study conducted by Dykes et al.^7^, who used the same method. Those authors found that the extravasation volume was 10-49 mL in 51% of the patients and 50-99 mL in 32% of the patients, both ranges being similar to those found in our study. That is probably related to the fact that our method of preventing extravasation followed the guidelines of care recommended worldwide^7^.

In our study, no statistical correlation was found between the extravasated volume and the type of venous catheter used. That is probably because, at each institution, the choice of venous catheter depends on the requirements of the CT scan^8^. In an evaluation of 118,970 contrast medium injections (ICM extravasation occurring in 289 patients), Moreno et al. found a significant correlation between smaller catheter caliber and lower mean extravasated volume, concluding that it is probably due to early detection of extravasation in smaller caliber catheters. Those authors also concluded that the mean volume of extravasated ICM is lower when the catheter is inserted in the radiology department immediately before the injection than when it is inserted in other departments or is left in place for more than 24 h^12^.

None of the patients in our study evolved to a serious complication; 94.10% had mild extravasation, and 5.90% had moderate extravasation. In 13.50% of our patients, additional clinical intervention, such as evaluation by the emergency department or vascular surgery, was required. In a study of 502,391 ICM injections, Shaqdan et al. evaluated patients who underwent tomography and magnetic resonance imaging. Of the 451 patients submitted to tomography, 35 (7.76%) were classified as having no damage, 415 (92.02%) were classified as having mild/temporary damage, and only 1 (0.22%) was classified as having major damage, the last progressing to blisters and ulceration, although not requiring surgical intervention^8^.

Dykes et al. also evaluated the degree of intensity and found that it was mild in 94.60% of their patients, moderate in 4.70%, and severe in 0.80%. Only one patient, a male with an extravasation volume of 150 mL, required surgery^7^. In the clinical evolution of extravasation, approximately 98% of patients require only conservative therapy, and the evolution to complications requiring surgical intervention is rare. In our sample, this was confirmed, and none of our patients presented severe complications after the event, nor did they require surgery or hospital admission for treatment of an ICM extravasation event.

The results of the present study should be considered in the context of some of its limitations. Because it was a retrospective study, it was not possible to evaluate the clinical and demographic data of all the patients who underwent CT, as those data were not available, so it was not possible to identify risk factors related to the incidence of extravasation. It was also not possible to evaluate factors related to the venipuncture technique, such as the number of puncture attempts, the department in which the puncture was performed, and the degree of experience of the technician or nurse who performed the procedure. In addition, the small number of cases impaired the analysis of potential risk factors for moderate/severe extravasation. We believe that future prospective studies, with standardized data collection, could allow a better analysis of these aspects.

In conclusion, the incidence rate of ICM extravasation in cancer patients submitted to CT was 0.20% in the present study, considered similar to the incidence in the general population, according to other studies found in the literature. The clinical, demographic, and oncological characteristics of the patients who presented contrast medium extravasation in the studied population were heterogeneous, demonstrating that any patient can present this type of complication. The vast majority of cases of extravasation were considered mild, and no severe cases were observed in the study sample. Additional clinical procedures, such as emergency department evaluation and vascular surgery, were required in 13.5% of extravasation cases, although no patient required hospitalization or surgery.
